# Exploring the Benefits of Extra Virgin Olive Oil on Cardiovascular Health Enhancement and Disease Prevention: A Systematic Review

**DOI:** 10.3390/nu17111843

**Published:** 2025-05-28

**Authors:** Sara Ussia, Giovanna Ritorto, Rocco Mollace, Maria Serra, Annamaria Tavernese, Carmen Altomare, Carolina Muscoli, Massimo Fini, Francesco Barillà, Ciro Indolfi, Pasquale Perrone Filardi, Vincenzo Mollace, Roberta Macrì

**Affiliations:** 1 Pharmacology Laboratory, Institute of Research for Food Safety and Health IRC-FSH, Department of Health Sciences, University Magna Graecia of Catanzaro, 88100 Catanzaro, Italy; saraussia1598@gmail.com (S.U.); giovanna.ritorto@studenti.unicz.it (G.R.); carmen.altomare@studenti.unicz.it (C.A.); muscoli@unicz.it (C.M.); mollace@libero.it (V.M.); roberta.macri@unicz.it (R.M.); 2 Department of Experimental Medicine, University “Tor Vergata” of Rome, 00133 Rome, Italy; francesco.barilla@uniroma2.it; 3 Department of Medicine and Surgery, University Campus Bio-Medico of Rome, 00128 Rome, Italy; an.tavernese@gmail.com; 4 Cardiology Department, IRCCS San Raffaele Roma, Via di Valcannuta 250, 00166 Rome, Italy; massimo.fini@sanraffaele.it; 5 Department of Pharmacy, Health and Nutritional Sciences, University of Calabria, Via Pietro Bucci, 87036 Rende, Italy; indolfi@hotmail.com; 6 Cardiology Department, University of Naples Federico II, 80138 Naples, Italy; pasquale.perrone@unina.it; 7 Renato Dulbecco Institute, 88046 Lamezia Terme, Italy

**Keywords:** virgin olive oil, cardiovascular disease, oxidative stress, inflammation, endothelial dysfunction, polyphenols, metabolic dysfunction, atherosclerosis

## Abstract

**Introduction:** Olive oil’s health benefits are widely known and extensively documented; its advantages are widespread, covering numerous areas of human health. Clinical and experimental data indicate that a Mediterranean Diet (MedDiet) with Extra Virgin Olive Oil (EVOO) lowers the risk of illnesses associated with oxidative stress, chronic inflammation, and weakened immunity, including cancer and cardiovascular disease (CVD). The European Food Safety Authority (EFSA) confirms that olive oil’s polyphenols help protect blood lipids against oxidative damage; thus, EVOO, crucial in the MedDiet, could be a functional food component. Olive oils must contain at least 5 mg of Hydroxytyrosol (HYTY) and its derivatives (oleuropein and Tyrosol (TY)) per 20 g to qualify for the EFSA-approved health claim. To provide a summary of clinical study results, this systematic review assessed the impact of Virgin olive oil (VOO) consumption on cardiovascular risk and disease prevention. **Methods:** The systematic review’s studies were collected from PubMed, Cochrane, Web of Science, and Scopus following the Preferred Reporting Items for Systematic Reviews and Meta-Analyses (PRISMA) statement and the Population Intervention Comparison Outcome Population (PICO) framework. **Results:** Seventeen clinical studies were identified, which highlighted the association between VOO consumption (including EVOO) and a reduced risk of cardiovascular disease. Particularly, improvements in biomarkers involved in cardiometabolic pathways and subsequent cardiovascular events were recorded. The beneficial effect was attributed to the polyphenols contained in EVOO. Indeed, EVOO supplementation as part of the Mediterranean diet could improve patients’ quality of life in secondary prevention by demonstrating a positive correlation with the cardioprotective role of polyphenols. **Discussion:** A balanced diet with VOO represents a simple yet potent method to counteract metabolic dysfunctions associated with CVD. Despite these results, further multicenter clinical studies with a wider range of patients are required to confirm and better understand EVOO’s effects on the prevention of cardiovascular risk.

## 1. Introduction

*Olea Europaea* L., commonly referred to as the wild Olive, is originally from the Mediterranean area and is regarded to be the ancestral arboreal species. Olive cultivation, harvesting, and oil extraction have historically been central to the Mediterranean diet in this area, providing olive oil and olives as a main food source [1].

The different potential impacts on human health of various olive oil sub-types, such as Extra Virgin Olive Oil (EVOO), Virgin Olive Oil (VOO), Refined Olive Oil (ROO), and olive pomace oil, were identified. Accordingly, supporting evidence suggests a crucial impact of olive oil in daily practices and cultural advancement since its earliest deployment in the 7th century AC [1]. The biomolecules contained in Olea Europaea L. extract are classified into saponifiable, triglycerides and detached fatty acids, with a proportion of more than 98–99%, and unsaponifiable (hydrocarbons, aliphatic, perfumed alcohols, tocopherols, aldehydes, fat-soluble vitamins, phenols, normal unsafe compounds, triterpenic acids, sterols, etc.) characterizing a small percentage [2]. Several factors impact the composition of this minor classification, including development area, olive variety, climate, product availability, processing methods, treatment type, and capacity needs, distinguishing EVOOs from lower-quality olive oils [2,3]. The European Food Safety Authority (EFSA) validated a health suggestion for polyphenols present in olive oil [4]. According to a welfare claim, founded on logical evidence validated ten years earlier, the EFSA in May 2012 certified that “polyphenols in olive oil contribute to the safety of blood lipids from oxidative stress”, and EVOO, the essential lipid component of the Mediterranean Diet (MedDiet), may be considered functional nutrient [5]. Specifically, phenolic compounds characterizing VOO are phenolic alcohols including Hydroxytyrosol (HYTY) and Tyrosol (TY), and their Secoiridoids derivatives oleuropein aglycon, oleuropein and oleocanthal [3,4]. The EFSA-approved health claim is only for olive oils with a minimum of 5 mg HYTY and its derivatives (oleuropein and TY) per 20 g. Accordingly, high phenolic EVOO (HP-EVO) is characterized by a minimum content of 250 mg of polyphenols/kg of oil, as defined by the standards criteria [6]. The definite polyphenols mentioned by EFSA are HYTY, TY, and further complex polyphenols of high molecular mass from which HYTY and TY (HYTY- and TY-related forms) can be derived, known as secoiridoids [7]. The health advantages of olive oil have been widely acknowledged and extensively documented [8]. Clinical, epidemiological, and experimental evidence shows that the Mediterranean diet, including extra virgin olive oil, reduces the risk of diseases linked to oxidative stress, chronic inflammation, and immune system issues like cancer, atherosclerosis, and cardiovascular disease (CVD) [9,10], helps to improve chronic liver diseases, like Metabolic Dysfunction-Associated Steatotic Liver Disease (MASLD) and liver fibrosis, reducing the likelihood of liver cancer [8]. In addition, it influences several factors associated with cardiovascular risk, such as lipoprotein profile, Blood Pressure (BP), glucose metabolism, and antithrombotic profile promoting endothelial function [11,12,13,14]. Therefore, this systematic review aims to report on the benefits of VOO consumption for CVD patients, focusing on improved cardiac biomarker levels.

## 2. Methods

### 2.1. Database Sources

This systematic review examines the correlation between VOO and CVD, according to EFSA guidelines, using data from randomized clinical trials (all phases) to assess the influence of a MedDiet rich in VOO such as dietary intervention to prevent cardiovascular risk and disease. Using the Preferred Reporting Items for Systematic Reviews and Meta-Analyses (PRISMA) statement and the Population Intervention Comparison Outcome (PICO), this systematic review selected studies from PubMed, Cochrane, Web of Science, and Scopus. All articles written in English and published between January 2005 and 2025 were included. For the qualitative analysis of this systematic review, only articles published within the last 20 years were selected. The review protocol was registered with Prospero (PROSPERO 2025 CRD420251029375) (Supplementary Material File S1).

### 2.2. Eligibility Criteria

Criteria for inclusion and exclusion are specified as follows: (a) human studies, (b) randomized clinical trial, (c) VOO and CVD, correspond to the inclusion criteria; furthermore, (a) meta-analysis, (b) review, (c) non-human, (d) animal experiment, (e) unrelated disease, (f) legume, (g) compared to other oil and nutrients, (h) vitro and vivo studies, (i) limitation, (j) not original studies, (k) not full text available, were chosen for exclusion criteria.

### 2.3. Study Outcomes

The consumption of VOO combined with pharmacological treatments may have beneficial effects linked to an improvement of CVD incidents in patients reporting high cardiovascular risk. Indeed, there is evidence suggesting EVOO may be cardioprotective, because of its considerable antioxidant phenolic content (PC). Particularly, polyphenols have been demonstrated to improve the antiatherogenic function of High-Density Lipoprotein (HDL), endothelial function, plasma high-density lipoprotein cholesterol (HDLc), plasma levels of oxidized Low-Density Lipoprotein (ox-LDL), lipid peroxide, Systolic Blood Pressure (SBP) in hypertensive patients. This systematic review assessed the effectiveness of VOO-supplemented diets on cardiovascular issues by analyzing clinical study results. In order to meet this objective, we investigated several clinical studies that collected the activities of High Polyphenol Olive Oil (HPOO) compared to Low Polyphenol Olive Oil (LPOO) or two similar olive oils but with a different polyphenol concentration (ROO, VOO) and evaluate the efficacy of a MedDiet supplemented with VOO compared to a low-fat diet (LF diet).

### 2.4. Statistical Analysis

Results from comparable exposure groups in different papers were qualitatively summarised.

## 3. Results

### 3.1. Data Collection

**Figure 1:**
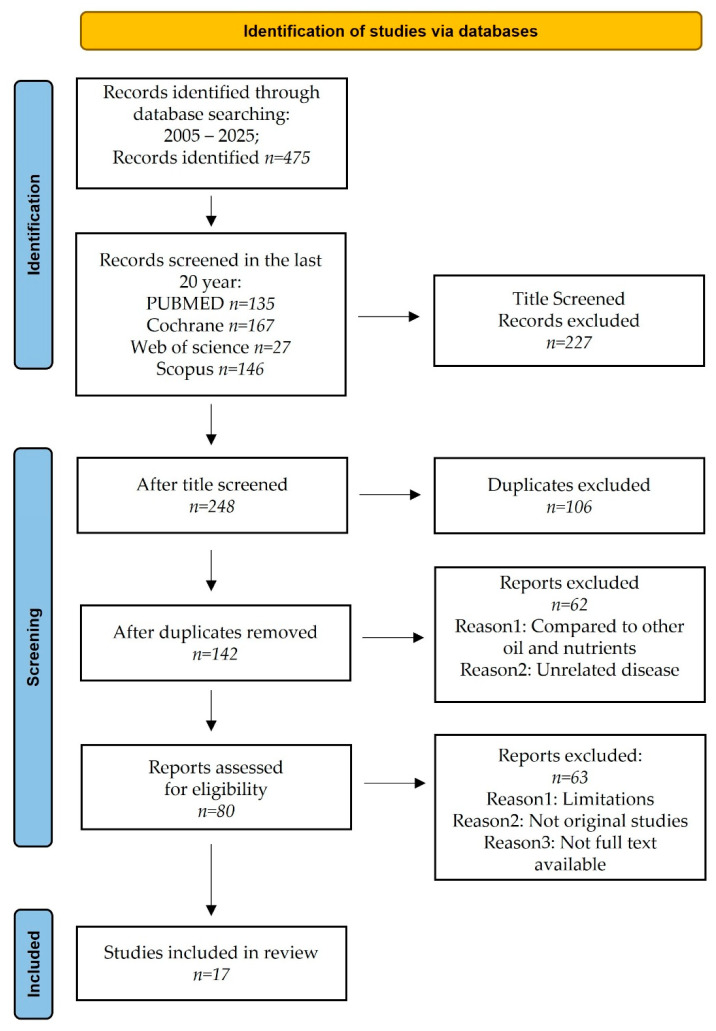
Prisma flow chart.

A literature search from 2005 to 2025 (conducted in January) identified a total of 475 records. After screening the titles, we identified 92 PubMed, 62 Cochrane, 18 Web of Science, and 76 Scopus. Following the removal of duplicates, 142 papers were selected for abstract screening; 62 were excluded based on keywords present in their abstracts and 80 full-text papers to be evaluated for eligibility. Based on the exclusion criteria, we excluded 63 articles because they concerned: limitations (low sample), not original study, full text not available, other oil, and unrelated disease. Finally, 17 papers were included in the qualitative analysis (Figure 1).

### 3.2. Virgin Olive Oil Supplementation and Cardiovascular Diseases

The main features of 17 on supplementation of VOO in patients suffering from cardiovascular disorders are summarized in Table 1.

**Figure 2:**
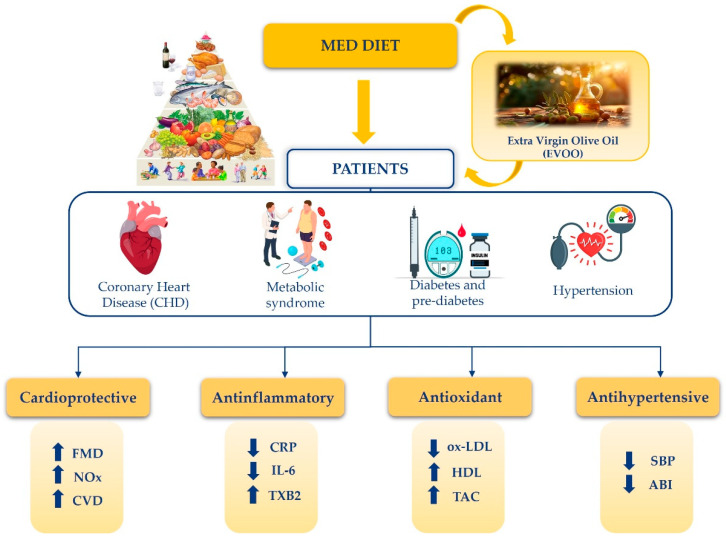
Beneficial effects of Mediterranean diet on patients suffering from coronary artery disease, metabolic syndrome, diabetes, pre-diabetes, and hypertension. Mediterranean diet, characterized by a high intake of extra virgin olive oil, had the most significant beneficial effect on cardiovascular health. Specifically, improvements in flow-mediated vasodilation and increased nitric oxide availability were observed, which contribute to a reduced risk of cardiovascular events. Additionally, decreased levels of C-reactive protein and interleukin-6, alongside increased thromboxane B2 concentrations, were evident, indicating a notable anti-inflammatory effect. In regard to protection against oxidative stress, the Mediterranean diet was found to significantly lower oxidized LDL, while enhancing HDL and total antioxidant capacity. Finally, the Mediterranean diet has shown potential antihypertensive effects, as evidenced by reductions in both systolic blood pressure and the ankle-brachial index. The arrows indicate decrease (↓) and increase (↑), respectively. Mediterranean Diet (MedDiet); Coronary heart disease (CHD); Extra virgin olive oil (EVOO); Flow-mediated dilation (FMD); Nitrogen Oxides (NOx); Cardiovascular Disease (CVD); C-reactive Proteine (CRP); Interleukin-6 (IL-6); Tromboxane B2 (TXB2); Oxidized low-density lipoprotein (ox-LDL); High-density lipoprotein (HDL); Total antioxidant capacity (TAC); Systolic blood pressure (SBP); Ankle-brachial index (ABI).

The reviewed studies reported that the consumption of EVOO plays a pivotal role in preventing recurrent cardiovascular events, emphasizing its essential role in integrated health interventions in addition to pharmacological treatment. The clinical trials showed a correlation between an improvement in cardiovascular risk factor parameters and dietary supplementation with EVOO, highlighting the strong antioxidant properties of its polyphenol content (Figure 2).

To improve the interpretation of the results, we categorized the data by age, classifying the patients into different groups and ordering them in order of age. Thus, we could differentiate between young and old patients. Specifically, the age groups include over 18 years; between 20 and 80 years; between 38 and 55 years; over 55 years; an average of 67.3 years; and over 80 years.

Evidence from in vitro studies highlights the regulatory influence of phenolic compounds in EVOO on oxygen-dependent enzymatic pathways. Specifically, hydroxytyrosol (HT) and oleuropein (OE) exhibit pronounced enzyme-modulating activity and function as potent free radical scavengers and antioxidants, thereby reinforcing the biochemical significance of EVOO as a reserve of bioactive molecules [15]. The objective of the study was to comparatively evaluate the vasoprotective potential of EVOO in patients with mild dyslipidemia. In a crossover study, 22 patients received 40 mL/day of either phenol-rich extra-virgin or phenol-poor refined olive oil. Each treatment was administered for a period of seven weeks. Notably, decreased serum TXB2 and increased plasma antioxidant capacity were observed in both treatment groups after EVOO administration. Circulating cardiovascular markers improve in mildly dyslipidemic patients who consume EVOO. These effects might be linked to cardioprotection, according to current understanding [15].

A study was carried out with the objective to investigate the association between consumption of olive oil and subclinical atherosclerosis, as well as the risk of total CVD, CHD, and stroke. Three cohorts were involved in the trial: The AWHS cohort (2318 men), the SUN Project (18,266 men and women), and the EPIC-Spain cohort (39,393 men and women). In the context of these Spanish studies, a higher consumption of total olive oil was found to be associated with a reduced risk of both CVD and stroke. The maximum benefit was observed with a consumption between 20 and 30 g/day. Virgin olive oil exhibits properties that potentially counteract the early formation of coronary and atheromatous plaques by positively influencing calcium deposits and reducing their density, which could have benefits for cardiovascular health. A multidetector-row CT scanner was used to obtain coronary calcium, which was then quantified using calcium scoring software in accordance with the Agatston method. The data suggested that VOO should be consumed in larger quantities and with greater frequency than other fats, ideally from early life onwards, for primary CVD prevention [16].

To assess the impact on cardiovascular events, a follow-up clinical study compared a VOO-rich Mediterranean diet to a low-fat diet in participants with initial CHD, observing outcomes after 7 years. The outcome included specific cardiovascular events: revascularization, myocardial infarction, documented peripheral arterial disease, ischemic stroke, and cardiovascular death. The Coronary Diet Intervention with Olive Oil and Cardiovascular Prevention (CORDIOPREV) included 1002 coronary patients from Spain from 2009–2012. In this population, 58% had metabolic syndrome: Participants in the LF diet group successfully lowered their fat intake from 36.7% to 32.1%, showing a 12.5% average reduction, surpassing similar intervention studies. Due to the high-intensity dietary intervention in both groups and participants’ strong adherence to healthy dietary models, along with optimal health treatment, a lower rate of cardiovascular events than expected was observed in the trial. In secondary prevention, the MedDiet was higher than the LF diet to prevent cardiovascular dysfunction [17].

An additional study was carried out to investigate if a long-term diet rich in olive oil or a low-fat diet could improve Endothelial dysfunction (ED) in the CORDIOPREV clinical trial, and whether the benefits were consistent across patients with or without T2D. According to the American Diabetes Association (ADA) criteria, the participants were divided into three groups: T2D patients, prediabetic individuals, and those without T2D. Compared to a low-fat diet, the Mediterranean diet improved flow-mediated dilation (FMD) in patients with type 2 diabetes or prediabetes; in those without diabetes, both diets maintained similar FMD levels. The evidence suggests habitual EVOO-rich MedDiet consumption improves endothelial function in prediabetic and diabetic patients [18].

Healthy volunteers consumed EVOO with different polyphenol concentrations; this trial monitored postprandial circulating microRNA changes and determined the metabolic pathways the altered microRNAs affected. This is a randomized, double-blind, parallel-group, post-meal study. Twelve healthy participants each consumed 30 mL of EVOO with varying phenol levels: 250 mg/kg (L-EVOO), 500 mg/kg (M-EVOO), and 750 mg/kg (H-EVOO). Quantitative real-time PCR analysis of plasma postprandial microRNA levels showed a decrease in let-7e-5p, miR-328a-3p, let-7e-5p, miR-10a-5p, miR-21-5p and miR-26b-5p and an increasing level of miR-17-5p, miR-20a-5p, miR-17-5p, miR-20a-5p and miR-192-5p following consumption of the three different oils. Therefore, the adjustment of post-meal circulating microRNA levels could potentially explain the cardiovascular advantages linked to consuming EVOO [19].

This study assessed the effects of phenolic and triterpenoid-rich VOO on metabolic syndrome and endothelial function biomarkers in healthy individuals. A three-week randomized, crossover, double-blind, controlled intervention study was conducted on 58 participants who received a daily dose (30 mL) of three oils. The analysis included a VOO, an OVOO, and a FOO with the following compositions: VOO (124 ppm phenolic compounds, 86 ppm triterpenes), OVOO (490 and 86 ppm, respectively), and FOO (487 and 389 ppm, respectively). The ingestion of a daily dose of extra virgin olive oil, which has been enriched with phenolic compounds, has been demonstrated to enhance the levels of HDL in the blood plasma. The study showed a decrease in endothelin-1 plasma levels after the consumption of the three olive oils, as well as in stimulated blood cell cultures. In particular, VOO with at least 124 ppm of phenolic compounds decreased the pre-intervention plasma levels of endothelin-1 regardless of triterpene content (*p* < 0.05) [20].

This clinical trial aims to assess how FVOO impacts endothelial function in patients with hypertension. In a double-blind, randomized, crossover postprandial trial, thirteen patients with pre-hypertension or stage 1 hypertension each received a single 30 mL dose of either FVOO (OOPC 961 mg/kg) or VOO (OOPC 289 mg/kg). FVOO showed higher IRH (*p* < 0.05) and plasma Cmax of HYTY sulphate, an OOPC metabolite, 2 h post-lunch compared to VOO (*p* = 0.05). After ingestion of FVOO, the ox-LDL decreased (*p* = 0.010) in an inverse relationship to the AUC values of IRH (*p* = 0.01). FVOO provided greater benefits on endothelial function than a standard natural VOO in pre- and hypertensive patients [21].

A further study examined the effects of HPOO versus LPOO on blood pressure and arterial stiffness in healthy Australian adults. A double-blind, randomized, controlled crossover study enrolled 50 participants (mean age 38.5 ± 13.9 years, 66% women) who received either 60 mL/day of HPOO (360 mg/kg polyphenols) or LPOO (86 mg/kg polyphenols) for three weeks.

HPOO supplementation showed a statistically significant reduction of 2.5 mmHg (95% CI: −4.7 to −0.3) and 2.7 mmHg (95% CI: −4.7 to −0.6) in peripheral and central SBP, respectively. Moreover, olive oil did not affect DBP or arterial stiffness measurements.

Reductions in SBP following HPOO consumption offer clinical backing for using HPOO as a dietary intervention against CVD in diverse groups [22].

A further study investigated how HPOO and LPOO affected HDL’s cholesterol efflux capacity in healthy adults. Fifty participants, aged between 38 and 55 years old (with a standard deviation of 13.9), 66% of whom were female, took part in a double-blind, randomized, cross-over study. Over a three-week period, their daily intake was either HPOO (320 mg/kg polyphenols) or LPOO (86 mg/kg polyphenols). The two groups receiving the same treatment did not show any significant differences in lipid levels or body measurements. Additionally, there were no meaningful variations in HDL cholesterol efflux in the LPOO and HPOO treatment groups; the respective values were 0.54% (95% CI (0.29, 1.37)) and 0.10% (95% CI (0.74, 0.94)). After intake of LPOO and HPOO, serum HDL increased significantly, by 0.13 mmol/L (95% CI (0.04, 0.22)) and 0.10 mmol/L (95% CI (0.02, 0.19), respectively. However, a moderate but significant increase in LDL of 0.14 mmol/L (95% CI (0.001, 0.28)), was observed after intervention with HPOO [23].

According to the cardiovascular health benefits of olive oil polyphenols, a randomized clinical trial evaluated the antioxidant and anti-inflammatory effects of extra-virgin polyphenol-high olive oil (HP- EVOO) compared to LPOO in healthy Australian adults.

In a double-blind cross-over trial, 50 subjects (age 38.5 ± 13.9 years, 66% female) were randomized to receive 60 mL/day of HPOO (320 mg/kg polyphenols) or LPOO (86 mg/kg polyphenols) for three weeks. Within the subgroup characterized by abdominal obesity, ox-LDL levels saw a reduction of 13.5 mU/mL (95% CI −23.5 to −3.6), while TAC levels increased by 0.04 mM (95% CI 0.006–0.07); within the inflammation subgroup, a 1.9 mg/L decrease in hs-CRP (95% CI −3.7 to −0.1) was observed exclusively in the HPOO group. The antioxidant and anti-inflammatory effects of HPOO are supported by improvements observed, particularly in high-risk adult populations. According to the strong correlation between oxidative stress and inflammation directly related to cardiovascular dysfunction, the results of this clinical trial highlight the potential preventive activity of extra virgin HPOO in healthy individuals with CVD [24].

The evaluation of another study suggests the effects of PC in VOO on endothelial reactivity in hypercholesterolemic patients. Twenty-one volunteers consumed two types of olive oil-based breakfasts: one rich in phenols and one with reduced PC. The phenol-rich breakfast improved endothelial function, increased NOx levels, and reduced LPO and 8-epi prostaglandin-F2α levels. Positive correlations were found between NOx and enhanced endothelial function, while negative correlations were observed between IRH and both LPO and 8-epi prostaglandin-F2α concentration. The antioxidant compounds in olive oil likely reduce oxidation and significantly increase NO metabolite levels, helping to prevent ED associated with acute fat intake [25].

There’s an inverse relationship between olive oil consumption and the risk of developing type 2 diabetes and cardiovascular disease. This study will find plasma metabolites linked to different olive oil uses and see how those metabolites relate to type 2 diabetes and cardiovascular disease risk. Baseline metabolomics data were available for 1837 participants at high cardiovascular risk in the PREDIMED trial, making up the discovery population. Large-scale epidemiological study findings show an inverse link between baseline metabolite profiles (total and EVOO) and CVD incidence (hazard ratio per standard deviation = 0.79). Conversely, there was no significant link found between the above-mentioned metabolite profiles and the development of T2D. Olive oil and extra virgin olive oil consumption has been associated with a reduced risk of cardiovascular disease in a high-risk Mediterranean population but not type 2 diabetes [26].

A study aimed to determine if different types of olive oil consumption correlated with ABI in PREDIMED-Plus participants. In a study of 4330 individuals, those in the top 20% of olive oil consumption showed a positive correlation with ABI (beta = 0.014, 95% CI: 0.002, 0.027, *p* = 0.010). Comparing olive oil consumption to ABI, higher virgin olive oil intake correlated inversely with low ABI (OR 0.73, 95% CI [0.56, 0.97]), while higher olive pomace oil intake correlated positively (OR 1.22, 95% CI [1.00, 1.48]). A Mediterranean population with increased cardiovascular risk showed a positive correlation between olive oil consumption and mean ABI. The results indicate a possible link between olive oil consumption and reduced peripheral artery disease risk, but longitudinal studies are needed to confirm this association [27].

A randomized, crossover, double-blind, placebo-controlled study was carried out based on dietary supplementation with the two olive oils containing different concentrations of phenolic compound (ROO:14.67, VOO:161 mg/kg). Participants received 50 mL of VOO and ROO daily for two 3-week periods, separated by 2-week ROO-only washout periods. Food intake was documented via a validated food frequency questionnaire during each intervention and inflammatory markers were evaluated. Urinary TY, HYTY, and O-methylhydroxytyrosol (MOHT), established as compliance biomarkers, were assessed by Gas Chromatography-Mass Spectrometry (GC-MS). According to the intervention with ROO, the VOO treatment decreased IL6 (*p* < 0.002) and CRP (*p* = 0.024) and increased T, TYHY and MOHT (*p* < 0.001). In conclusion, consuming VOO for 3 weeks resulted in a higher reduction of IL6 and CRP than after consumption of ROO, in patients suffering from stable CHD. No alterations in soluble intercellular and vascular adhesion molecules, glucose, and lipid profile were reported. Finally, VOO could supplement drug treatment to positively impact patients with stable coronary artery disease [28].

Another study showed that two comparable olive oils had different levels of PC (refined: 14.7 mg/kg vs. virgin: 161.0 mg/kg) and demonstrated antioxidant and antihypertensive effects in 40 men with stable CHD. A study was conducted using two olive oils with different PC concentrations (ROO with 14.67 mg/kg and VOO with 161 mg/kg) in a randomized, crossover, placebo-controlled design. The combination of VOO and ROO was used in two sets of 3-week periods, with 2-week breaks in between where only ROO was given. During the intervention, participants consumed 50 mL of olive oil daily, divided into three doses. Decreased plasma levels of ox-LDL (*p* < 0.001) and lipid peroxide (*p* = 0.003) were reported after VOO, combined with increased glutathione peroxidase activity (*p* = 0.033). Hypertensive patients showed a significant SBP decrease (*p* = 0.001) following VOO administration, particularly those with baseline SBP ≥ 140 mmHg. The trial demonstrated the potential benefit of using PC-enriched VOO as an adjunct to pharmacological treatment in CHD patients [29].

Diet, particularly fat intake, plays a pivotal role in the inflammatory response associated with the development of atherosclerosis, regardless of fasting status. This study examines the effect of dietary fat after meals on the expression of genes associated with inflammation (e.g., NF-κB, MCP-1, TNF-α, and IL-6) and plaque stability (e.g., MMP-9) in peripheral blood mononuclear cells (PBMCs). Twenty healthy seniors participated in a three-week dietary study, each following a different diet. (1) Med Diet enriched in Monounsaturated Fatty Acid (MUFA) with virgin olive oil; (2) SFA-rich diet; and (3) low fat, CHO-PUFA diet by a randomized crossover design. Fasting and eating a Mediterranean diet resulted in significantly less p65 NF-kB gene expression than fasting and eating a high-SFA diet (*p* = 0·019). Compared to SFA-rich and CHO-PUFA diets, the Mediterranean diet lowered postprandial gene expression of p65, MCP-1, MMP-9, and TNF-α (*p* values ranged from 0.0229 to 0.047). In elderly individuals, a Mediterranean diet appears to lessen the inflammatory response after meals in mononuclear cells, unlike diets high in saturated fat and carbohydrate-PUFA [30].

The latest randomized controlled trial investigated how following a MedDiet impacts blood pressure and endothelial function in older, healthy Australians. 166 participants aged 64+ were randomly assigned to follow either a Mediterranean diet (85 participants) or their usual diet (81 participants) for six months. Participants who followed the MedDiet showed a significant reduction in SBP compared to the control group that followed the usual diet. Endothelial function, assessed by FMD, is improved in the MedDiet group at 6 mo, the percentage of FMD was higher by 1.3% in the MedDiet group.

The article suggests that adherence to a MedDiet can bring significant benefits to cardiovascular health, helping to reduce BP and improve blood vessel function [31].

## 4. Discussion

This systematic review provides a report on the advantageous effects of VOO supplementation in patients suffering from CVD, with a particular emphasis on the improvement of biomarkers implicated in cardiac pathways.

The results collection from the last 20 years (2005–2025) offers a broader perspective on the results obtained, providing a clinical evaluation of nutraceutical supplementation in combination with pharmacological treatment to ameliorate the quality of life of patients. Indeed, the outcome of clinical studies highlighted the crucial cardioprotective activity of Olive Oil supplementation [14].

CVD has been identified as a prevalent condition within the general population; several dysfunctions are involved in CVD development, such as high BP, lipid abnormalities, increased inflammatory biomarkers profiles, chronic inflammatory conditions, ED, and oxidative stress [32]. From traditional medicine, olive oil consumption has played a pivotal role in the Mediterranean population. Moreover, the protective properties of olive oil, particularly EVOO, have been extensively documented in several studies which have emphasized its potential in primary and secondary CVD prevention, T2D, and other chronic conditions [6,33].

CHD is a chronic condition characterized by a progressive narrowing of the blood vessels supplying the myocardium with oxygenated blood. This results in periods of oxygen deprivation during periods of increased oxygen demand [34].

The consumption of VOO, enriched with polyphenols, could have beneficial effects in patients with CHD as an adjunctive and complementary primary prevention to pharmacological treatment [35]. Indeed, higher consumption of total olive oil produced a lower risk of CVD and stroke; in particular, optimal benefits are seen when consuming 20–30 g daily [16].

Additionally, the adherence to the MedDiet in secondary prevention has been supported by additional investigations with remarkable clinical outcomes [17].

Interestingly, central hemodynamic parameters have been proven to be meaningful predictors of cardiovascular (CVD) events and all-cause mortality [36].

Hemodynamic changes, like peripheral (arm) and central (aortic) blood pressure, are linked to the occurrence of harmful cardiovascular events. In addition, the stiffening of central elastic arteries is an independent, indirect predictor of CVD risk; it also shows a positive association with systolic hypertension [22].

A growing number of studies have provided evidence that consumption of HPOO results in a significant decrease in both peripheral and central SBP in healthy adult subjects from Australia. However, no alterations in DBP or arterial stiffness measures were observed [22,37].

The role of novel inflammatory markers in the diagnosis and prognosis of CVD has been well established [38]. Several evidence identified a correlation between different pro-inflammatory cytokines and multiple instances of CVD, often categorized as pro-inflammatory states (e.g., IL-6, TNFα, and interleukin-1 (IL-1) increase) [39,40]. In particular, EVOO may exert an influence on genes that are linked to inflammation and atherosclerotic plaque stability [30]. Effectively, in a crossover design experiment, olive oil was found to have a significant positive effect on inflammatory markers in the blood. This impact was characterized by a reduction in serum TXB2 production, suggesting a potential anti-inflammatory role for olive oil [15]. Furthermore, it was highlighted that HPOO, in comparison to ROO, is more efficacious in decreasing CRP and IL-6 levels in stable CHD patients [28].

Additionally, there’s an inverse relationship between HDL plasma levels and CVD incidence, and we found that HDL cholesterol efflux capacity inversely correlates with CVD incidence [20]. Thereby, some studies focusing on metabolic syndrome in healthy adults showed that olive oil consumption increased plasma HDLc and exhibited the HDL capacity to remove cholesterol [23].

EVOO is a key element of the MedDiet and has protective effects on the cardiovascular system, preventing atherosclerosis onset and progression [14]. The polyphenols in EVOO exert anti-thrombotic and anti-inflammatory activity, improve the bioavailability of nitric oxide, and reduce BP. These effects translate into a reduced risk of atherosclerotic plaque formation and CVD such as hypertension and CHD [9]. Therefore, the inclusion of EVO oil in the daily diet could represent an effective strategy for cardiovascular risk reduction [41]. Particularly, ox-LDL has been shown to have a significant role in the development and progression of atherosclerotic plaque formation [24]; confirming this, low levels of ox-LDL and endothelin-1 were observed after supplementation with olive oil, which could ensure a reduced development of the atherosclerotic phenotype [24].

Finally, the process of oxidative damage can be prevented or reduced through the action of different endogenous or exogenous antioxidant scavengers. In consideration of this issue, it has been observed a correlation between plasma TAC, an indicator of the body’s overall antioxidant status, and a decreased risk of developing chronic diseases, including CVD [24]. Furthermore, a study showed that olive oil’s antioxidant compounds are able to determine TAC improvement, hs-CRP levels, and oxidative stress reduction with enhanced nitric oxide metabolites, helping to prevent ED associated with acute fat intake and the Med diet increased FMD [24,25].

The outcome of many scientific research studies highlighted that EVOO more significant impact on the modulation of physiological parameters than other foods and oils [42]. Its bioactive properties are a combination of polyphenols and monounsaturated fatty acids, contributing to its effectiveness as a dietary supplement when consumed as part of a balanced diet [43].

Notably, a particular study demonstrated that replacing conventional, predominantly refined olive oil with phenol-rich extra virgin olive oil, results in a cholesterol-reducing effect unrelated to the fatty acid content. This suggests that the active compounds in EVOO have a beneficial impact on cardiovascular health in addition to the basic lipid profile [44].

Another research carried out on individuals suffering from high cardiovascular risk provides additional support for this claim. A lower incidence of major cardiovascular events has been demonstrated among those following a Mediterranean diet enriched with EVOO or dried fruit, compared to individuals following a reduced-fat dietary regimen. These findings emphasize the potential of EVOO supplementation as a promising and substantiated approach for the prevention of cardiovascular disease [45].

Finally, a marked distinction is evident in the correlation of EVOO with other dietary fats, as in the case of butter. The saturation and unsaponifiable fraction of these two lipids are different, but extra virgin olive oil, which is rich in polyphenols and other minor components, is beneficial to health, especially by improving cardiometabolic parameters [46].

Ultimately, extra virgin olive oil is not only a crucial component of the Mediterranean diet but also a highly recommended approach to encourage cardiovascular and metabolic health.

Further research is crucial to establish the specific role of EVOO bioactive compounds in cardiovascular health, particularly concerning their effects on gene expression, cellular signaling, and enzymatic functions [47]. Accordingly, the formulation of precisely characterized and standardized olive oil-based dietary supplements, with clearly published compositional data, would facilitate future research aimed at identifying the specific molecular constituents underlying their cardioprotective efficacy [42].

The latest models of circular economy, which promote eco-friendly technologies for the recovery of active compounds from by-products and waste, are already employed in the olive oil industry. Indeed, the development of study methods for the innovative use of fractions standardized in HT content, as precursors for the synthesis of new biologically active molecules with rich bioavailability, has been achieved through the assessment of the biological and biomedical activities of many secondary metabolites of *Olea europaea* L. [48].

The improvement of the quality and efficacy of EVOO is significantly impacted by technological advancements. The enhancement of extraction techniques, the guarantee of sustainable production practices, and the safety and authenticity of EVOO products are promising prospects for further investigation [49]. Lastly, producing nutraceutical EVOO-based products could be a valuable opportunity. These products concentrate on specific bioactive compounds and enhance bioavailability achieved by using advanced delivery systems, such as liposomes and nanoparticles [50].

To investigate the impact of different olive oil capsule formulations using innovative and pragmatic trial designs focused on low-cost methodologies could be a realistic approach to exploring this prominent topic [42].

The cardiovascular benefits of EVOO could be increased, and healthcare costs reduced by enhancing people’s knowledge of how to select and use it appropriately, according to scientific evidence. This could be achieved by establishing clear innovative guidelines to include it as part of a diet designed to protect heart health [49].

## 5. Conclusions

In conclusion, this systematic review analyzed the supplementation of VOO, specifically EVOO, in relation to cardiovascular dysfunction, highlighting its contribution to the prevention of CVD and showing the beneficial activity of EVOO polyphenols to counteract cardiovascular damage.

Indeed, EVOO intake might improve protection against inflammation, oxidative stress, blood clotting, high blood pressure, endothelial function, and lipid dysfunctions, thus modulating several factors predisposing to cardiovascular events.

The integration of olive oil in a nutritionally balanced diet represents a simple yet powerful strategy for promoting heart health. This statement emphasizes the pivotal role of dietary selection in preventing chronic illnesses and enhancing overall well-being.

Nevertheless, additional multicenter clinical studies, which enroll a wider range of patients are needed to validate and support this evidence and to clarify the potential beneficial consequences of EVOO consumption.

## Figures and Tables

**Table 1:** The table shows the selected articles, specifying the clinical trial aims, databases used, and results.

Authors, Year	Aim of Studies	Types of Studies Included	Summary of Results	Ref.
Visioli F, et al., 2005	Assess the vasoprotective potential of EVOO in slightly dyslipidemic patients.	The randomised study followed a crossover design.	↓ serum Thromboxane B2 (TXB2) levels ↑ plasma antioxidant capacity ↑ Cardiovascular markers	[15]
Donat-Vargas C, et al., 2022	Examine if the consumption of olive oil is related to subclinical atherosclerosis, the risk of total CVD, CHD and stroke.	Randomised clinical trial	↓ CVD and stroke risk ↓ creation of heart artery calcium and plaques.	[16]
Delgado-Lista J, et al., 2022	Evaluate the efficacy of a MedDiet supplemented with VOO compared to a LF diet in impacting the composite prevalence of cardiovascular events.	A randomised, single blind, controlled dietary intervention trial.	↓ total amount of fat ↓ cardiovascular event rate	[17]
Torres-Peña JD, et al., 2018	Compare the effectiveness of a high-fat Mediterranean di-et (rich in olive oil) versus a low-fat diet in preventing cardiovascular events and death among patients with a history of CHD, using a long-term follow-up.	An ongoing prospective, randomised, single blind, controlled trial including 1002 patients with CHD.	↑ Flow-Mediated Dilation (FMD) and endothelial function	[18]
Daimiel L, et al., 2020	Explore postprandial modifications in circulating microRNA levels after acute intake of three phenol-enriched EVOO to establish the metabolic pathways targeted by the modified micro Ribonucleic Acid (microRNAs).	A randomised, postprandial, parallel, double-blind study.	↓ level of let-7e-5p, miR-328a-3p, let-7e-5p, let-7e-5p, miR-10a-5p, miR-21-5p and miR-26b-5p ↑ levels of miR-17-5p, miR-20a-5p, miR-17-5p, miR-20a-5p and miR-192-5p.	[19]
Sanchez-Rodriguez E, et al., 2018	Estimate the influence of VOO, rich in phenolic compounds and triterpenoids, on metabolic syndrome and biomarkers of endothelial function in healthy adults.	A three-week randomised, crossover, controlled, double-blind, intervention study.	↑ Plasma HDLc ↓ Plasma endothelin-1 levels	[20]
Valls RM, et al., 2015	Evaluate the effects of EVOO on endothelial function in hypertensive patients.	A randomised, double-blind, crossover postprandial study.	↓ ox-LDL ↑ endothelial function	[21]
Sarapis K, et al., 2020	Determine the differences in blood pressure and arterial stiffness between HPOO and LPOO groups of healthy Australian adults.	A double-blind, randomised, controlled cross-over trial.	↓ peripheral SBP	[22]
Sarapis K, et al., 2023	Examine how HPOO and LPOO affect HDL’s ability to enhance cholesterol elimination in healthy adults.	A double-blind, randomised cross-over trial.	↑ Serum HDL ↑ LDL levels of 0.14 mmol/L	[23]
Sarapis K, et al., 2022.	Examined the antioxidant and anti-inflammatory impact of HPOO in comparison to LPOO in healthy Australian adults.	A double-blind cross-over, randomised trial.	↓ ox-LDL ↑ TAC ↓ high-sensitivity C-Reactive Protein (hs-CRP)	[24]
Ruano J, et al., 2005.	Investigate the impact of the PC of VOO on endothelial reactivity in hypercholesterolaemic patients.	A randomised sequential crossover design.	↑ endothelial function and NOx ↓ LPO and 8-epi prostaglandin-F2alpha	[25]
García-Gavilán JF, et al., 2023	This study aims to discover plasma metabolites associated with various olive oil in-takes and explore their links to type 2 diabetes and CVD risk.	Clinical trial	↓ CVD incidence	[26]
Sánchez-Quesada C, et al., 2020	Analyze the association between the intake of edible olive oils and Ankle-Brachial Pressure Index (ABI) in participant of the PREDIMED-Plus study.	A cross-sectional analysis of the PREDIMED-Plus trial.	↓ ABI	[27]
Fitó M, et al., 2008	Assess how different phenolic compound concentrations in two olive oils affect inflammatory markers in stable coronary artery disease patients.	A placebo-controlled, crossover, double-blind, randomised trial.	↓ Interleukin-6 (IL-6) and C-reactive protein (CRP) ↑ T, TYHY and MOHT	[28]
Fitó M, et al., 2005	Determine the antioxidant and antihypertensive effects of two olive oils (refined: 14.7 mg/kg polyphenols; virgin: 161.0 mg/kg polyphenols) in a study of 40 men with stable CHD.	A placebo controlled, crossover, randomised trial.	↓ ox-LDL and lipid peroxide ↑ glutathione peroxidase activity ↓ SBP following VOO intake.	[29]
Camargo A, et al., 2012	Estimate the efficacy of dietary fats on the expression of genes related to inflammation and plaque stability during the postprandial state of twenty healthy and elderly subjects.	A randomised, crossover design.	↓ post-meal gene expression of p65, Mono-cyte Chemoattractant Protein 1 (MCP-1), and Matrix Metallo-proteinase-9 (MMP-9) ↓ gene expression of p65 and Tumor Necrosis Factor-a (TNF-α)	[30]
Davis CR, et al., 2017	Establish the effects of adherence to the consumption of a MedDiet diet on BP and endothelial function in healthy elderly Australians.	A randomised controlled trial.	↓ SBP ↑ Endothelial function	[31]

The arrows indicate decrease (↓) and increase (↑), respectively. ABI: ankle-brachial index; CHD: Coronary heart disease; CRP: C-reactive protein; CVD: cardiovascular disease; EVOO: Extra Virgin Olive Oil; FMD: Flow-mediated dilation; HDLc: high-density lipoprotein cholesterol; HPOO: high polyphenol olive oil; LDL: low-density lipoprotein; LF diet: low-fat diet; LPO: lipoperoxides; LPOO: low polyphenol olive oil; MCP-1: monocyte chemoattractant protein 1; microRNAs: Micro ribonucleic acid; MMP-9: matrix metalloproteinase 9; NO(x): nitric oxide metabolites; Ox-LDL: oxidized low-density lipoprotein; PC: phenolic content; TAC: Total antioxidant capacity; TNF-a: tumor necrosis factor-a; TXB2: Thromboxane B2; VOO: Virgin Olive Oil.

## Supplementary Materials

The following supporting information can be downloaded at: https://www.mdpi.com/article/10.3390/nu17111843/s1, File S1: Prospero (PROSPERO 2025 CRD420251029375. Reference [51] is cited in the supplementary materials.

## Abbreviations

ABI	ankle-brachial index.
ADA	American Diabetes Association.
AUC	area under the curve.
BP	blood pressure.
CHD	Coronary heart disease.
CHO-PUFA	high-carbohydrate diet enriched in *n*-3 PUFA.
CI	confidence interval.
Cmax	maximum observed concentration.
CORDIOPREV	Coronary Diet Intervention with Olive Oil and Cardiovascular Prevention.
CRP	C-reactive protein.
CVD	cardiovascular disease.
DBP	Diastolic BP.
ED	Endothelial dysfunction.
EFSA	The European Food Safety Authority.
ELISA	enzyme-linked immunosorbent assay.
EVOO	Extra Virgin Olive Oil.
FFQ	food-frequency questionnaires.
FMD	Flow-mediated dilation.
FOO	functional olive.
FVOO	functional virgin olive oil.
GC-MS	Gas Chromatography Mass Spectrometry.
H-EVOO	high extra virgin olive oil.
HabDiet	habitual diet.
HDL	High-Density Lipoprotein.
HDLc	high-density lipoprotein cholesterol.
HP-EVO	high-phenolic Extra Virgin Olive Oil.
HPOO	high polyphenol olive oil.
HR	Hazard Ratio.
hs-CRP	high-sensitivity C-reactive protein.
HYTY	HYDROXYTYROSOL.
IL-1	interleukin-1.
IL6	Interleukin-6.
IRH	Ischemic reactive hyperemia.
L-EVOO	low extra virgin olive oil.
LC–MS	liquid chromatography-mass spectrometry.
LDL	low-density lipoprotein.
LF diet	low-fat diet.
LPO	lipoperoxides.
LPOO	low polyphenol olive oil.
M-EVOO	medium extra virgin olive oil.
MASLD	Metabolic Dysfunction-Associated Steatotic Liver Disease.
MCP-1	monocyte chemoattractant protein 1.
MedDiet	Mediterranean Diet.
microRNAs	Micro ribonucleic acid.
MMP-9	matrix metalloproteinase 9.
MOHT	O-methylhydroxytyrosol.
MUFA	Monounsaturated Fatty Acids and Health.
NF-kB	nuclear factor kappa-light-chain-enhancer of activated B cells.
NO(x)	nitric oxide metabolites.
OOPC	own phenolic compounds.
OR	odds ratio.
OVOO	optimised Virgin Olive Oil.
Ox-LDL	oxidized low-density lipoprotein.
PC	phenolic content.
PICO	Population Intervention Comparison Outcome Population.
PREDIMED	Prevention with Mediterranean Diet.
PRISMA	Preferred Reporting Items for Systematic Reviews and Meta-Analyses.
ROO	refined olive oil.
SBP	systolic blood pressure.
SFA	saturated fatty acid.
sICAM-1	Soluble Intercellular Adhesion Molecule-1.
sVCAM-1	Soluble Vascular Cell Adhesion Molecule-1.
T2D	Type 2 diabetes.
TAC	Total antioxidant capacity.
TAG	triacylglycerol.
TNF-a	tumor necrosis factor-a.
TXB2	Thromboxane B2.
TY	tyrosol.
VOO	Virgin Olive Oil.
