# Supplementary material for: Exploring the Benefits of Extra Virgin Olive Oil on Cardiovascular Health Enhancement and Disease Prevention: A Systematic Review
Source: Nutrients. 2025 May 28;17(11):1843. doi: 10.3390/nu17111843 (PMC12158199; doi:10.3390/nu17111843)
Supplement: Supplementary file 1 [file nutrients-17-01843-s001.zip › nutrients-3618122-supplementary.pdf]

NIHR | National Institute for Health and Care Research

PROSPERO
International prospective register of systematic reviews

Exploring the Benefits of Extra Virgin Olive Oil on Cardiovascular Health Enhancement and Disease Prevention: A Systematic Review

Roberta Macrì, Vincenzo Mollace, Rocco Mollace, Giovanna Ritorto, Sara Ussia, Maria Serra

Citation

Roberta Macrì, Vincenzo Mollace, Rocco Mollace, Giovanna Ritorto, Sara Ussia, Maria Serra. Exploring the Benefits of Extra Virgin Olive Oil on Cardiovascular Health Enhancement and Disease Prevention: A Systematic Review. PROSPERO 2025 CRD420251029375. Available from https://www.crd.york.ac.uk/PROSPERO/view/CRD420251029375.

REVIEW TITLE AND BASIC DETAILS

Review title

Exploring the Benefits of Extra Virgin Olive Oil on Cardiovascular Health Enhancement and Disease Prevention: A Systematic Review

Condition or domain being studied

Cardiovascular disease; Olive Oil; Oxidative stress; Inflammation; Endothelial dysfunction; Polyphenols; Metabolic Disease; Atherosclerosis

Rationale for the review

Olive oil's health benefits are widely known and extensively documented; advantages are widespread, covering numerous areas of human health. Clinical and experimental data indicate that a Mediterranean diet with extra virgin olive oil lowers the risk of illnesses associated with oxidative stress, chronic inflammation, and weakened immunity, including cancer and cardiovascular disease. The EFSA confirms that olive oil's polyphenols help protect blood lipids against oxidative damage; thus, EVOO, crucial in the Mediterranean diet, could be a functional food component. Olive oils must contain at least 5mg of HYTY and its derivatives (oleuropein and TY) per 20g to qualify for the EFSA-approved health claim. To provide a summary of clinical study results, this systematic review assessed the impact of Virgin olive oil consumption on cardiovascular risk and disease prevention.

Review objectives

This systematic review aims to report on the benefits of VOO consumption for cardiovascular disease patients, focusing on improved cardiac biomarker levels,through the amelioration of oxidative stress, inflammation, endothelial and metabolic dysfunction and atherosclerosis.

Keywords

Virgin olive oil; Cardiovascular disease; Oxidative Stress; Inflammation; Endothelial Dysfunction; Polyphenols; Metabolic dysfunction; Atherosclerosis

Country

Italy

ELIGIBILITY CRITERIA

Population

Included

(a) human studies, (b) randomized clinical trial, (c) virgin olive oil and cardiovascular disease,

Excluded

(a) meta-analysis, (b) review, (c) non-human, (d) animal experiment, (e) unrelated disease, (f) legume, (g) compared to other oil and nutrients, (h) vitro and vivo studies, (i) limitation, (j) not original studies, (k) not full text avaiable

Intervention(s) or exposure(s)

Included

Nutrient supplementation; Olive Oil; Cardiovascular System; Antioxidants; Antiinflammatory Agents; Lipid-lowering diet

Excluded

Trials in which EVOO supplementation was combined with other functional foods, such as nuts, coconut oil and palm oil, were excluded.

Comparator(s) or control(s)

This review does not have any comparators

Study design

Only randomized study types will be included.

Included

human studies, randomized clinical trial

Excluded

non-human, animal experiment, unrelated disease, legume,compared to other oil and nutrients, in vitro and in vivo studies

Context

Diet virgin olive oil supplementation in human studies

TIMELINE OF THE REVIEW

Date of first submission to PROSPERO

09 April 2025

Review timeline

Start date: 13 January 2025. End date: 2 April 2025.

Date of registration in PROSPERO

09 April 2025

AVAILABILITY OF FULL PROTOCOL

Availability of full protocol

A full protocol has not been written.

SEARCHING AND SCREENING

Search for unpublished studies

Only published studies will be sought.

Main bibliographic databases that will be searched

The main databases to be searched are *PubMed* and *Scopus*.

Other important or specialist databases that will be searched

Web of science

Search language restrictions

The review will only include studies published in English.

Search date restrictions

Databases will be searched for articles published from 13 January 2005 and before by 13 January 2025.

Other methods of identifying studies

No other methods will be used.

Link to search strategy

A full search strategy is not available.

Selection process

Studies will be screened independently by at least two people (or person/machine combination) with a process to resolve differences.

Other relevant information about searching and screening

None

DATA COLLECTION PROCESS

Data extraction from published articles and reports

Data will be extracted independently by at least two people (or person/machine combination) with a process to resolve differences.

Authors will not be contacted for further information.

Study risk of bias or quality assessment
Risk of bias/study quality will not be assessed.

Reporting bias assessment
Risk of bias due to missing results will not be assessed

Certainty assessment
Certainty of findings will not be assessed

OUTCOMES TO BE ANALYSED

Main outcomes
Cardiovascular disease counteraction; oxidation and inflammation decrease; metabolic and endothelial dysfunction amelioration; LDL decrease. The consumption of virgin olive oil combined with pharmacological treatments may have beneficial effects linked to an improving of CVD incidents in patients reporting high cardiovascular risk. Indeed, there is evidence suggesting extra virgin olive oil (EVOO) may be cardioprotective, partly due to its high phenolic content (PC) with antioxidant properties. Particularly, polyphenols have been demonstrated to improve the antiatherogenic function of HDL, endothelial function, plasma high-density lipoprotein cholesterol (HDLc), plasma levels of oxidized LDL, lipid peroxide, Systolic blood pressure in hypertensive patients. The aim of this systematic review was to investigate the efficacy of diet supplemented with virgin olive oil in influencing the composite prevalence of cardiovascular events and dysfunctions through clinical studies to provide an overview of the results. To achieve this objective, we examined several clinical studies that collected the effects of high polyphenol olive oil (HPOO) compared to low polyphenol olive oil (LPOO) or two similar olive oils but with a different polyphenol concentration (ROO, VOO) and evaluate the efficacy of a Mediterranean diet supplemented with virgin olive oil compared to a low-fat diet.

Additional outcomes
There are no additional outcomes.

PLANNED DATA SYNTHESIS

Strategy for data synthesis
No formal data synthesis is planned - data will be described but not combined.

CURRENT REVIEW STAGE

Stage of the review at this submission

Review stage	Started	Completed
Pilot work	✓	✓
Formal searching/study identification	✓	✓
Screening search results against inclusion criteria	✓	✓
Data extraction or receipt of IPD		
Risk of bias/quality assessment		

Review stage	Started	Completed
Data synthesis		

Review status
The review is currently planned or ongoing.

Publication of review results
Results of the review will be published in English.

REVIEW AFFILIATION, FUNDING AND PEER REVIEW

Review team members
Dr Roberta Macrì (review guarantor and contact) ORCID: 0000-0002-2345-6751. University Magna Graecia of Catanzaro. Italy.

No conflict of interest declared.

Professor Vincenzo Mollace. University Magna Graecia of Catanzaro,. Italy.

No conflict of interest declared.

Professor Rocco Mollace. University Magna Graecia of Catanzaro. Italy.

No conflict of interest declared.

Dr Giovanna Ritorto. University Magna Graecia of Catanzaro,. Italy.

No conflict of interest declared.

Dr Sara Ussia. University Magna Graecia of Catanzaro,. Italy.

No conflict of interest declared.

Dr Maria Serra. University Magna Graecia of Catanzaro,. Italy.

No conflict of interest declared.

Named contact
Dr Roberta Macrì (robertamacri85@gmail.com). ORCID: 0000-0002-2345-6751. University Magna Graecia of Catanzaro. Italy.

Review affiliation
Magna Graecia University of Catanzaro, Italy

Funding source
Review has no specific/external funding but is supported by guarantor/review team (non-commercial) institutions.

Peer review
There has been no peer review of this planned review.

ADDITIONAL INFORMATION

Review conflict of interest
Declared individual interests are recorded under team member details.. No additional interests are recorded for this review.

Medical Subject Headings

Atherosclerosis; Biomarkers; Cardiovascular Diseases; Humans; Inflammation; Olive Oil; Oxidative Stress

SIMILAR REVIEWS

Check for similar records already in PROSPERO

PROSPERO identified a number of existing PROSPERO records that were similar to this one (last check made on 9 April 2025). These are shown below along with the reasons given by that the review team for the reviews being different and/or proceeding.

- Impacts of regular extra virgin olive oil dietary consumption on oxidative stress and inflammation: a systematic review and meta-analysis [published 4 April 2022] [CRD42022314584]. The review was judged **not to be similar**
- Olive oil consumption can reduce the risk of Cardiovascular disease: a meta-analysis [published 1 November 2023] [CRD42023413242]. The review was judged **not to be similar**
- Biomarkers of Periodontitis and Their Association with Cardiovascular Disease: A Systematic Review and Meta-Analysis [published 5 July 2024] [CRD42024561810]. The review was judged **not to be similar**
- Oleocanthal, phenolic compound in Extra Virgin Olive Oil (EVOO): A Comprehensive Systematic Review on Its Potential in Inflammation and Cancer [published 17 November 2023] [CRD42023479713]. The review was judged **not to be similar**
- Oleocanthal, phenolic compound in Extra Virgin Olive Oil (EVOO): A Comprehensive Systematic Review on Its Potential in Inflammation and Cancer [published 17 November 2023] [CRD42023477093]. The review was judged **not to be similar**
- The Role of Advanced Lipoproteins and Novel Biomarkers in Forecasting Cardiovascular Events in Asymptomatic Populations: A Systematic Review [published 19 November 2024] [CRD42024611894]. The review was judged **not to be similar**
- The effects of extra virgin olive oil on blood pressure of hypertensive and pre-hypertensive patients: a systemic review and meta-analysis [published 25 January 2019] [CRD42019120713]. The review was judged **not to be similar**
- A protocol for systematic review and meta-analysis of effects of Yoga on oxidative stress and antioxidant capacity in people with cardiovascular disease or increased risk [published 25 October 2022] [CRD42022367135]. The review was judged **not to be similar**
- Systemic inflammation, oxidative stress and cardiovascular health in children and adolescents: a systematic review [published 4 May 2022] [CRD42022316807]. The review was judged **not to be similar**
- The clinical utility of biomarkers for cardiovascular risk assessment in children and young people growing up with HIV infection [published 29 October 2015] [CRD42015027675]. The review was judged **not to be similar**
- Association of Hydrocarbon Exposure and Risk of Cardiovascular Diseases and Cardiovascular Mortality: A Systematic Review and Meta-Analysis [published 13 December 2024] [CRD42024621372]. The review was judged **not to be similar**
- How to balance the benefits on cardiovascular outcome and the risk on rising glucose levels of Simvastatin as a secondary prevention treatment among patients with coronary

heart disease: systematic review and meta-analysis based on RCTs and observational studies [published 23 January 2018] [CRD42018081329]. The review was judged **not to be similar**
- Olive oil consumption and cardiovascular disease, cancer, and all-cause mortality: a systematic review and dose-response meta-analysis of prospective cohort studies [published 1 May 2023] [CRD42023419001]. The review was judged **not to be similar**
- Effects of Phytosterols on Cardiovascular Risk Factors: A Systematic Review and Meta-Analysis. [published 24 March 2024] [CRD42024522181]. The review was judged **not to be similar**
- Investigating the Ginseng Supplementation on Cardiovascular Disease Risk Factors: a Systematic Review and Meta-analysis [published 2 October 2023] [CRD42023465688]. The review was judged **not to be similar**
- Circulating proangiogenic cells for diagnosis and prognosis in cardiovascular disease: a review [published 24 June 2015] [CRD42015023717]. The review was judged **not to be similar**
- Effect of grape products on the levels of oxidative stress and inflammatory markers in patients with cardiovascular disease: a systematic review and meta-analysis [published 28 August 2024] [CRD42024579332]. The review was judged **not to be similar**
- Effects of vitamin E supplementation on endothelial function, inflammation and oxidative stress markers in hemodialysis patients: a systematic review and meta-analysis of randomized controlled trials. [published 25 July 2021] [CRD42021262773]. The review was judged **not to be similar**
- The effect of high-polyphenol olive oil on cardiovascular and metabolic risk factors: a systematic literature review and meta-analysis [published 21 June 2017] [CRD42017070060]. The review was judged **not to be similar**
- The effect of microecological preparations on renal function and oxidative stress and inflammation in patients with chronic kidney disease: a randomized controlled trial umbrella meta-analysis [published 17 March 2025] [CRD420251009842]. The review was judged **not to be similar**

PROSPERO version history

- Version 1.0, published 09 Apr 2025

Disclaimer

The content of this record displays the information provided by the review team. PROSPERO does not peer review registration records or endorse their content.

PROSPERO accepts and posts the information provided in good faith; responsibility for record content rests with the review team. The guarantor for this record has affirmed that the information provided is truthful and that they understand that deliberate provision of inaccurate information may be construed as scientific misconduct.

PROSPERO does not accept any liability for the content provided in this record or for its use. Readers use the information provided in this record at their own risk.

Any enquiries about the record should be referred to the named review contact
